# Industrial Internet of Things over 5G: A Practical Implementation

**DOI:** 10.3390/s23115199

**Published:** 2023-05-30

**Authors:** José Meira, Gonçalo Matos, André Perdigão, José Cação, Carlos Resende, Waldir Moreira, Mário Antunes, José Quevedo, Ruben Moutinho, João Oliveira, Pedro Rendeiro, Pedro Oliveira, Antonio Oliveira-Jr, José Santos, Rui L. Aguiar

**Affiliations:** 1 Fraunhofer Portugal AICOS, 4200-135 Porto, Portugal; 2 Bosch Termotecnologia SA, 3800-627 Cacia, Portugal; 3 Department of Electronics, Telecommunications and Informatics, Instituto de Telecomunicações, University of Aveiro, 3810-193 Aveiro, Portugal; 4 Instituto de Telecomunicações, 3810-193 Aveiro, Portugal; 5 Institute of Informatics, Federal University of Goiás, Goiânia 74690-900, Brazil

**Keywords:** 5G IoT end device, industrial 5G network, intelligent assistant, practical implementation

## Abstract

The next generation of mobile broadband communication, 5G, is seen as a driver for the industrial Internet of things (IIoT). The expected 5G-increased performance spanning across different indicators, flexibility to tailor the network to the needs of specific use cases, and the inherent security that offers guarantees both in terms of performance and data isolation have triggered the emergence of the concept of public network integrated non-public network (PNI-NPN) 5G networks. These networks might be a flexible alternative for the well-known (albeit mostly proprietary) Ethernet wired connections and protocols commonly used in the industry setting. With that in mind, this paper presents a practical implementation of IIoT over 5G composed of different infrastructure and application components. From the infrastructure perspective, the implementation includes a 5G Internet of things (IoT) end device that collects sensing data from shop floor assets and the surrounding environment and makes these data available over an industrial 5G Network. Application-wise, the implementation includes an intelligent assistant that consumes such data to generate valuable insights that allow for the sustainable operation of assets. These components have been tested and validated in a real shop floor environment at Bosch Termotecnologia (Bosch TT). Results show the potential of 5G as an enhancer of IIoT towards smarter, more sustainable, green, and environmentally friendly factories.

## 1. Introduction

Industrial premises make a complex system comprising various technologies distributed throughout an entire campus. By connecting the different assets (e.g., sensors, devices, machines) present on the shop floor to the industrial network, it is possible to collect a wide variety of factory data (e.g., air quality, vehicle travel routes, noise, movements, energy consumption, location, among others). The collected data are then processed to extract information that the management personnel of the factory can use to make informed decisions. These decisions can improve industrial operations’ efficiency, safety, and security, making them more cost-effective and increasing overall productivity. This entire process of connecting industrial assets on the shop floor is called Industrial IoT (IIoT) ( a complete list of abbreviations is available before the document references). Typical applications of IIoT include predictive maintenance, asset tracking, and energy management.

Throughout the evolution of industrial networks, most communication solutions were developed as dedicated applications without much focus on interoperability [1]. The lack of interoperability is a significant issue that threatens the predicted economic value of IoT in industrial settings [2]. It also leaves an opening for developing solutions that connect every asset in an industrial environment to the same network. The proposed 5G IoT end device aims to fulfil this opening by providing retrofittable, non-intrusive sensing and actuation capabilities, targeting different requirements of use cases in the industrial environment.

Moreover, the fifth generation of mobile broadband communication is seen as an enabler of IIoT and further encourages its adoption, as 5G allows for the transmission of large amounts of data with lower latency and higher reliability over secure links [3], while also having the flexibility necessary to adapt its communication performance in accordance to the industrial requirements. As industry communications are often critical for the correct operation of the industrial premises as a whole, they have strict requirements in terms of security and isolation, and a 5G non-public network (NPN) can answer such requirements while deploying an industrial 5G network. However, due to costs, regulation, or personnel expertise, every industry is expected to have different requirements for deploying an industrial 5G network. As such, different NPN deployment strategies were conceived that trade deployment and maintenance costs for security, isolation, and control over the network. The designed deployments range from stand-alone non-public network (SNPN) to PNI-NPN with different degrees of dedicated resources [4].

The proposed Industrial 5G Network is part of the 5GAIner laboratory. In this case, the network was set up to recreate a PNI-NPN network with a shared radio and control plane. To accomplish this, the 5G standalone (5G SA) core deployed at Instituto de Telecomunicações in Aveiro (ITAV) served as the operator core network. The industrial network uses the radio equipment deployed in the factory, which is connected to a factory multi-access edge computing (MEC) with an local breakout (LBO) user plane function (UPF). The MEC logically separates the industrial traffic from the public traffic, forwarding the industrial traffic to the industrial NPN and the public traffic to ITAV’s network.

From the application perspective, both the 5G IoT end device and industrial 5G network can be used for various purposes. In the scope of this work, these infrastructure components are complemented with an application component, the intelligent assistant. This assistant uses the data from the shop floor, gathered through Bosch TT’s data management framework, to make predictions and to consequently detect anomalies based on those predictions. Thanks to the capabilities of the 5G network, this assistant can use data collected according to a specific key performance indicator (KPI) (e.g., low-latency, high-reliability) and can provide predictions, allowing us to understand the behaviour of different assets on the shop floor.

With that in mind, this paper presents a practical implementation of IIoT over a 5G NPN whose contributions range from the infrastructure to the application levels and can be summarised as follows:

- A 5G IoT end device that is retrofittable and provides the means to seamlessly and non-intrusively actuate and sense different aspects of various industrial assets as well as their surrounding environment;
- A 5G network deployed on the shop floor, supporting the industrial NPN, while the communication performance is tailored to the specific needs of the target industrial use cases;
- An intelligent assistant that collects and processes real-time data aiming to make future behaviour predictions and to detect anomalies;
- A practical implementation of the proposed infrastructure and application components that serves as a baseline and provides lessons learned for future deployments of IIoT over a 5G NPN.

The practical implementation and validation of the proposed infrastructure and application components were realised at the Bosch TT’s premises as part of the Augmented Humanity (Augmanity- https://www.augmanity.pt/) project, which envisions improving the industrial working environments in different topics, such as workers’ health and safety, improved productivity, and improved efficiency in maintenance and energy usage.

The paper is structured as follows. Section 2 presents the works from other groups related to the proposed infrastructure and application components, emphasising existing efforts towards practical implementations. Section 3 highlights the different use cases that define the requirements to which our components must respond, while Section 4 presents our infrastructure and application components and how they work together towards building smarter, more sustainable, green, and environmentally friendly factories. Section 5 provides a collection of tests we have performed to validate the feasibility and functionality of the different components of our practical implementation. Finally, Section 6 concludes the paper with a general discussion of our findings while working towards the proposed IIoT over 5G practical implementation.

## 2. Related Work

End devices, sensing and actuating on the shop floor, are essential components of a 5G-enabled IIoT implementation. However, despite the potential of 5G, most of the proposals for industrial end devices either rely on other wireless communication protocols or exploit the perks of 5G through non-Third Generation Partnership Project (3GPP) access networks [5].

Malik et al. [6] developed a framework that connects non-destructive evaluation sensor data with real-time processing algorithms on an IoT system using both Wi-Fi and Bluetooth low energy (BLE). These are also the communication protocols selected by Lesjak et al. [7] and Nguyen et al. [8] on the proposed IIoT end devices. The authors in [9] present an IoT infrastructure substrate that provides real-time monitoring in multiple school buildings using IEEE 802.15.4 as the communication protocol. A survey on the trade-offs for using wireless networks in industrial applications is performed in [10], providing a baseline for the current state of industrial networks without 5G.

Cheng et al. [11] and Lin et al. [12] focus their work on 5G and IIoT by discussing the architecture and future possibilities of using 5G in IIoT scenarios. Both studies highlight the need for edge-computing in industrial 5G networks that can be done in an end device, but none proposes an end device. The disparity of performance between 5G SA and 5G non-standalone (5G NSA) is studied in [13,14], with the latter more focused on indoor environments and showing some similarity between 5G SA and 5G NSA when it comes to performance, with better throughput in 5G NSA but better overall latency for 5G SA. Despite exploring and testing different architectures for a 5G network in an industrial environment, these works are based on either network analysis or simulation without proposing and implementing an actual, deployable solution.

Focusing on 5G-enabled end devices, the authors in [15] explore a hardware-based on-demand sensing mechanism that aims to eliminate the energy waste caused by a sensor working in sleep mode. On the other hand, Verde et al. [16] use augmented reality to present an assistive maintenance framework connected to a 5G network via customer-premises equipment (CPE). Nevertheless, although proposing an end device with the required sensorisation for the identified use case, none of these solutions explore the use of 5G new radio (5G NR) for industrial applications using NPNs.

Regarding industrial 5G networks, each industry has different requirements and resources. For example, 5G Alliance for Connected Industries and Automation (5G-ACIA) [4] and 3GPP [17] identified different 5G deployment scenarios that range from installing dedicated 5G networks (SNPN) to deploying a virtual private network in the operator’s network in a specific network slice (PNI-NPN); some of them are identified in [4]. The obvious option to consider is a dedicated standalone 5G network for a particular vertical SNPN. However, this is not a cost-effective option in many cases since the vertical would have to acquire both equipment and a spectrum and would also have high maintenance costs. Alternatively, the 5G network’s support for network slicing is being leveraged to create logically isolated NPNs on top of public infrastructures (PNI-NPN). Moreover, MEC is being explored to provide the means for an LBO, redirecting 5G traffic to local servers and keeping data away from the public network while providing computing power near the end-users, reducing the communication latency. Thus, PNI-NPN, in conjunction with MEC LBO, realising delimited area slices and complemented by a user portal for provisioning and configuration, can be a good compromise for industries to deploy their digitisation process. The authors in [18,19] make a more profound analysis of the usage of NPN deployments in Industry 4.0. The former analyzes the NPN deployments, considering each deployment option’s benefits and constraints, while the latter focuses on analysing the deployment of NPN and the remaining challenges.

Regarding the intelligent assistant, during the last few years, many authors started exploring its potential in industrial environments. Peres et al. [20], for instance, propose a conceptual architecture that allows for a very efficient data-gathering process, as well as visualises and processes the gathered data. On a more advanced level, Coelho et al. [21] present an architecture where they implement an assistant capable of predictive maintenance tasks on sensorised stamping press, already with some practical results. Finally, Pravin et al. [22] propose a system that shows the potential of intelligent assistants towards industry and energy consumption. This system, applied to a production environment, uses machine learning algorithms to make a day-ahead forecast of some energy-related parameters, such as equipment energy demand and photovoltaic energy production. It then generates an optimised energy dispatch schedule that minimizes operational costs and carbon emissions. Despite these efforts, most of these solutions are still conceptual, with very few practical implementations.

## 3. Use Cases and Requirements

For the work’s scope, we focus on two use cases related to predictive maintenance and energy management. Based on these use cases, we collected the requirements that drove the development of the infrastructure and application components presented in Section 4.

### 3.1. Retrofit and Predictive Maintenance Use Case

This use case focuses on legacy assets installed at the customer premises (e.g., a boiler) and/or industrial assets on the shop floor (e.g., air compressors). A device, hooked to the asset, is able to sense and actuate on such an asset and communicates the sensed data to the manufacturer’s network. The sensing device must not interfere (i.e., non-intrusive) with the operation of the assets and cannot consume too much power. The data are used to understand the functioning of the asset and may serve the purpose of predictive maintenance processes.

Considering this, the sensing device must be modular and provide interfaces to support different sensors that help diagnose the machine’s health status. Temperature, pressure, humidity, vibration, energy, and air leaks are examples of readings from such sensors. The sensed data are then sent, over message queuing telemetry transport (MQTT), to a data repository and are processed to identify the behaviours that may lead to future machine failures. Automated alarms should be generated in such situations to trigger proactive maintenance procedures of assets, thus reducing maintenance downtime and associated costs.

For deploying this use case, a network must ensure the transmission of the sensor’s data to the data repository while providing reliable and energy-efficient communication. The network should also be capable of dispatching high volumes of data and ensure that the sensed data must not leave the manufacturer’s premises.

### 3.2. Energy Management Use Case

This use case aims to build energy consumption profiles of industrial assets (i.e., air compressors) based on data collected from attached sensing devices. Considering this, such devices need to have the capability of gathering different data related to the assets’ energy consumption. Sensing data are sent to a repository where they are saved and processed. The objective is to help to trigger commands (i.e., actuation) and alerts when energy consumption is anomalous or outside the desired boundaries. MQTT is the expected protocol for communication.

As the number of industrial assets to be monitored on the shop floor is high, this use case requires a network with reliable communication to support many connections, and that copes with the high volume of produced data traffic travelling from the shop floor to the data repository. With a focus on energy management, all the components used in this use case should be built with energy efficiency in mind.

### 3.3. System Requirements

Table 1 summarises the main requirements extracted from the aforementioned use cases and considered for developing our infrastructure and application components.

Considering the listed requirements, it is possible to conclude that the use cases share a common goal and non-conflicting requirements, allowing them to be built on the same system infrastructure. Both use cases consider a shop floor sensing device with a 5G NR interface that collects different data about industrial assets. The device must be non-intrusive by means of a modular and retrofittable design accompanied by an adaptive enclosure, which should expose the device’s connectors and sensors to guarantee access to the expansion ports and reliable sensor readings. In addition, it should not add an energy consumption burden to assets.

Data are exchanged over a reliable and energy-efficient 5G network between the shop floor and back-end operations. Once on the server side, the data are processed through a fault prediction mechanism to predict the behaviour, detect anomalies on the shop floor assets, and raise alerts or trigger commands that prompt proactive maintenance and energy management procedures.

The proposed solution comprises infrastructure and application components, which collect, transport, and process shop floor data and manage sensing devices. Section 4 details each component and how they meet the listed requirements.

## 4. Infrastructure and Application Components

**Figure 1:**
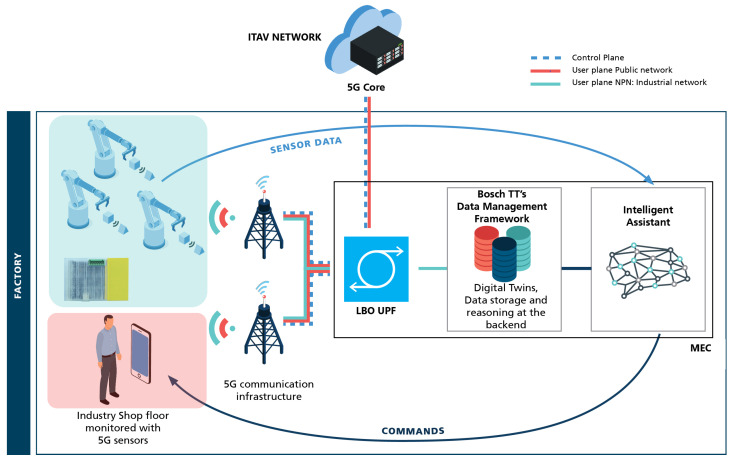
Our envisioned IIoT ecosystem over 5G.

Figure 1 provides a schematic representation of the whole IIoT ecosystem operating over 5G. It illustrates how our proposed infrastructure and application components work together towards a practical IIoT solution deployed over a 5G NPN implementation, aiming at building smarter, more sustainable, green, and environmentally friendly factories. It is noted that (i) this ecosystem is complete, with the proposed components duly tested and validated (cf., Section 5), and (ii) the work scope of this paper revolves solely around an industrial 5G NPN which works with a 5G IoT end device to collect data from industrial assets in the shop floor, as we are interested in understanding the behaviour of such a pure 5G system in an industrial setting that shall characterise the full-fledged system yet to be deployed in production at Bosch TT.

From the infrastructure perspective, different IoT devices sense and actuate on shop floor assets. Other user equipment (UE) (i.e., smartphone) can also be present on the shop floor. Sensed data, actuation commands, and UE traffic are sent over the 5G network.

The industrial part of this network is deployed at the factory and is connected to the core network of ITAV. This architecture is based on a NPN deployment that ensures the desired isolation by not letting industrial data leave the factory premises. The MEC with an LBO UPF redirects the traffic in a way to ensure that the UE, as a public user, has only access to the public network (continuous red line), while industrial data are only sent to the industrial network (continuous green line). Application-wise, the ecosystem allows the intelligent assistant to produce relevant energy management and maintenance information based on the sensed data, enabling data-driven, intelligent decision making. Next, we present the practical implementation of our proposed infrastructure and application components.

### 4.1. 5G IoT End Device

**Figure 2:**
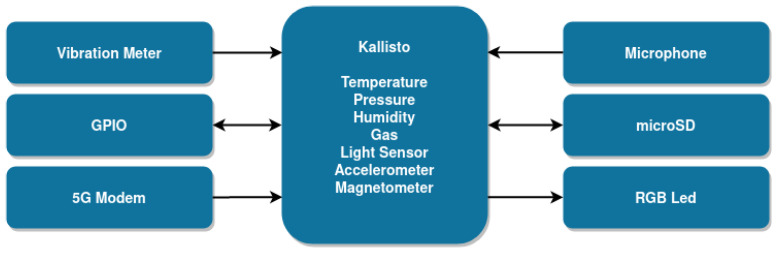
High-level hardware architecture of the 5G IoT end device.

The main goal of the end device is to collect data from industrial assets and the surrounding environment and to send them to a main server connected through 5G NR. Figure 2 presents the high-level architecture of its hardware components. The processing unit is the Kallisto^®^ IoT platform [23], which is equipped with an nRF52840 from Nordic [24] running the Zephyr OS [25]. Kallisto^®^ also comes with a set of sensors useful for data acquisition in industrial environments, namely: temperature, humidity, pressure, gas, and light exposure.

To be further aligned with the use cases identified in Section 3, we also added to the end device a vibration meter and a microphone to measure noise levels, as well as two input ports and two output ports for actuation in external devices. A set of general purpose input/output (GPIO) is also available, which allows instantiating both serial peripheral interface (SPI) and inter-integrated circuit (I^2^C) interfaces for connecting external components (e.g., proximity, infrared, and pH sensors or even another temperature sensor to measure temperature data in a different location from the device). An RGB led is also available to indicate the end device status at runtime.

The end device can be powered either by a universal serial bus type-c (USB-C) or a battery. It has a battery charge management circuit enabling battery charging when both the universal serial bus (USB) and battery are plugged into the board, as well as microSD card support for backup and non-volatile storage.

The support for 5G is added with a 5G modem connected to the Kallisto^®^ via an universal asynchronous receiver–transmitter (UART). The selection of this modem and the 5G antenna was one of the core steps of the development process because it determines the connection quality.

As of 2021, the market for 5G modems was still recent, and few solutions were available. The modem that covered most of the use case requirements identified in Section 3 was the RG502 from Quectel [26], as it supported 5G network and allowed for communication with our system-on-chip (SoC) via UART. However, this modem did not have built-in stack support for the transport layer and above, which was a requirement for the project.

Since the modem supported point-to-point protocol (PPP), a possible solution was to integrate the network stack on the SoC side. PPP is a communication protocol that is used in the data link layer that allows for direct communication between two devices, and it is designed to work with some network layer protocols [27], such as Internet protocol (IP). Both RG502 and nRF52840 support PPP over UART; thus, we could integrate the PPP stack layer over the UART connection based on the Zephyr OS global system for mobile communications (GSM) modem driver and then the IP layer over PPP. The remaining network stack was integrated into the nRF52840 using Zephyr OS implementation of the MQTT application layer protocol.

Additionally, at the time when this hardware was planned and developed, there were no available 5G SA nodes for public usage in Portugal. Having this in mind, long-term evolution (LTE) fallback was crucial for the development of the 5G IoT end device so that the firmware development and the full end-device validation could be performed in environments without 5G SA.

Considering this, the antenna had to at least support both LTE and 5G bands. We wanted an embedded antenna for the device, as it would improve the overall robustness and compactness of the whole design. In the end, the choice was the ACR4006X from Abracon [28]. This antenna provides ultra-wideband coverage and supports operating frequencies in the range of 600 MHz to 6 GHz. It also has decent gain values in the LTE Band 3 and 5G n78 bands, as well as good rejection in the 2.4 GHz (Wi-Fi, BLE) frequencies.

**Figure 3:**
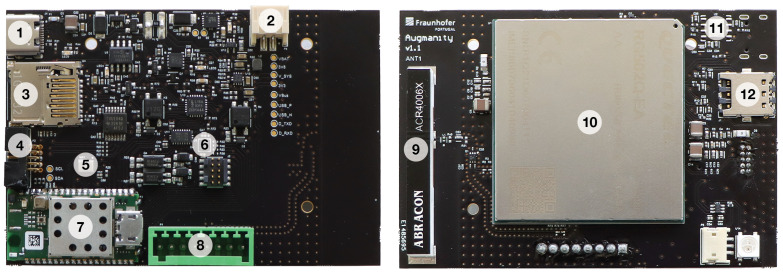
PCB and main components of the 5G IoT end device.

Figure 3 presents the developed printed circuit board (PCB) of the 5G IoT end device, highlighting its relevant components: (1) USB-C power and data connector; (2) external battery power connector; (3) microSD socket; (4) external I^2^C/SPI socket; (5) noise sensor—ICS43434 microphone [29]; (6) vibration meter—Kionix KX134 [30]; (7) Kallisto SY-020 module [23]; (8) sensor/actuator connector—two dry outputs, two wet inputs, 3.3V and GND pins available; (9) Abracon ACR4006X 600 MHz–6 GHz embedded antenna [28]; (10) RG502QEA 5G module [31]; (11) eSIM socket (for future versions of the end device); and (12) nanoSIM socket.

The concept design for the end device 3D-printed protective enclosure also followed the use case requirements identified in Section 3. Besides protecting the end device’s electronics, an important requirement for the enclosure was to guarantee access to the external connectors (e.g., USB-C, battery, microSD, I^2^C/SPI bus, GPIO) and sensors, such as the microphone used for noise collection. Another requirement for the concept design related to the fixation of the end device in assets (e.g., the exterior of a compressor) without requiring structural changes. After several tests, the following options were identified: (i) double-sided adhesive tape; (ii) magnet; (iii) fastening tape with a ratchet; and (iv) Velcro, with this last option being chosen.

**Figure 4:**
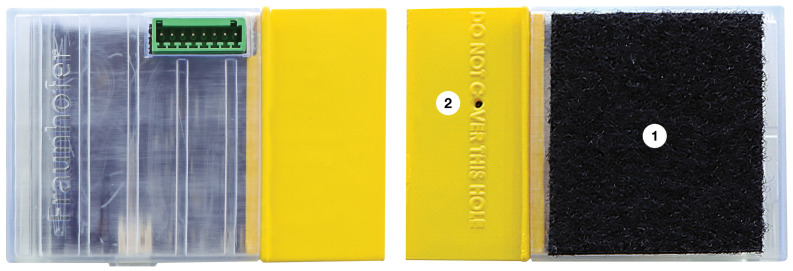
Three-dimensionally printed protective enclosure of the 5G IoT end device.

Figure 4 shows the enclosure, which has two halves, one in yellow, a colour commonly used on the shop floor to call attention to the presence of sensitive equipment. The other translucent part is intended to provide a better view of the interior of the end device. On one of the faces of the translucent part is the Velcro for industrial use (1). The Velcro area is about 70% of the area of the enclosure. Hence, we guarantee a strong fixation, and at the same time, if there is a vibration on the sensed asset, it is absorbed by the Velcro, and the collection of data is not compromised. As seen in Figure 4, the microphone (2) is located on a dedicated face with a recess to ensure that it will never be covered, in addition to a warning message not to cover the sensor’s access hole.

We have not stress tested the enclosure under extreme operating conditions, as its actual goal was to protect the electronics of the 5G IoT end device under normal, controlled environmental conditions and to allow for its proper fixation to industrial assets in the shop floor.

### 4.2. Industrial 5G Network

The industrial 5G network was deployed at the Bosch TT’s factory in Cacia, Aveiro, and was connected to a 5G core deployed at the ITAV, Portugal. For the deployment of this 5G network, the network needed to comply with all the Bosch TT’s security and isolation requirements while also complying with Portugal’s regulations. Since Portugal’s regulations only allow operators to own spectrum, it was necessary to deploy a 5G PNI-NPN network in the factory. Bosch TT’s isolation requirements also demand that all data must not leave factory premises. Considering these restrictions, the 5GAIner infrastructure was used, which is a 5G SA network that deploys the PNI-NPN network in Bosch TT’s factory. 5GAIner is presented and initially analysed in [32].

**Figure 5:**
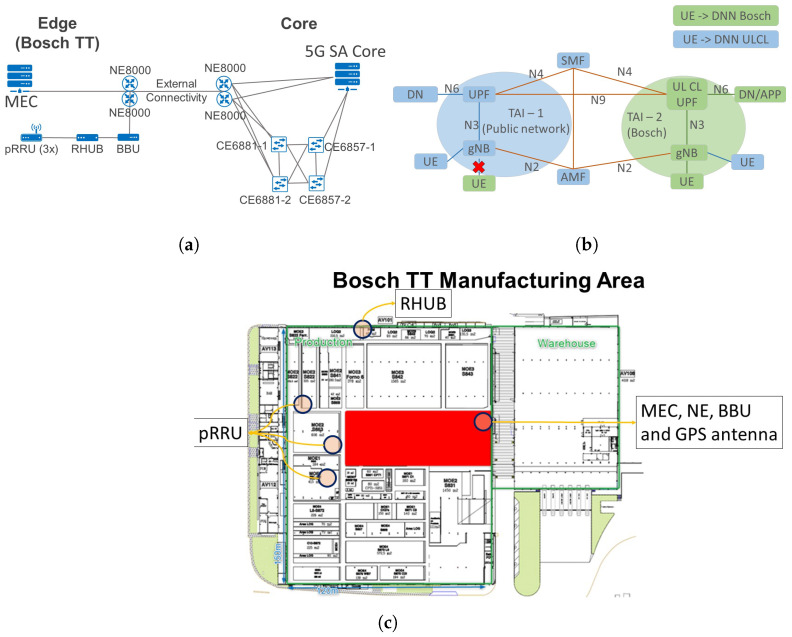
Industrial 5G network overview. (**a**) 5G Architecture overview. (**b**) Schematic of edge deployment at Bosch TT. (**c**) Network deployment locations at Bosch TT.

Figure 5a presents the overall architecture of the 5G architecture, identifying the different resources and their interconnections. The Bosch TT site is represented on the left side, including the radio and MEC solutions. Automatic neighbouring relationships have been set up to provide seamless handovers across the shop floor. On the right side, the core of the network is deployed at the ITAV premises, along with the supporting networking fabric providing communication support for the NFV infrastructure (NFVI) where the 5G functions are instantiated. The NFVI is monitored through the 5G network management platform, which orchestrates and manages the entire 5G network.

The Bosch TT factory’s infrastructure comprises a MEC LBO solution and a radio access network (RAN) solution with three pico remote radio unit (pRRU)s (indoor radio cells). The MEC solution was deployed to use a UPF that steers the end device’s data to the destination server or UE’s traffic to the public network. This ensures that all data produced from devices connected to the antennas deployed in the Bosch TT factory are directed to a local server without the need to pass through the 5G core. This guarantees high isolation from the public network by confining all industrial traffic to the factory premises, fulfilling Bosch’s requirements in terms of isolation. In addition, it improves the network’s performance in terms of security and latency by reducing the distance between the end device and the server.

Figure 5b details the interaction between the different 5G functions. The blue elements represent core deployments, whereas the green ones represent Bosch TT deployments. The local breakout functionality is based on an uplink classifier (ULCL) deployment, which helps to steer the traffic at the Bosch TT’s premises. ULCL is a UPF functionality that aims at diverting uplink traffic, based on filter rules provided by session management function (SMF), towards Data Network [33]. For this deployment, a different local area data network (LADN) was used for MEC, with a distinct data network name (DNN) and tracking area (TA). LADN and TA divide the radio network into sections, and in conjunction with DNN, only the allowed UEs can access the network in the Bosch TT radio network section. Therefore, the MEC network is identified by a different DNN and tracking area identifier (TAI) than the ones used in the core network, which ensures complete network isolation in the data plane between both sites. The DNN and TAI are used to select the UPF serving a given UE. Thus, this configuration ensures that only ITAV UEs can access the ITAV UPF and that Bosch TT UEs can access Bosch TT UPF, not allowing ITAV UEs to access Bosch TT UPF (only being able to use the control plane), and Bosch TT UEs cannot access the ITAV network at all.

The 5GAIner infrastructure was designed considering the requirements of the envisioned use cases to be supported. We deployed the following network functions (NFs) in the infrastructure: authentication server function (AUSF), access and mobility management function (AMF), SMF, network repository function (NRF), network slice selection function (NSSF), and unified data management (UDM) in the control plane. Two UPFs were deployed in the user plane, one deployed in the 5G core at ITAV, and the other in MEC at the Bosch TT factory. Figure 5c presents the physical deployment in the Bosch TT factory, which is divided into three physical locations: (i) a data center where the MEC, NetEngines and baseband unit (BBU) are placed; (ii) a rack on the shop floor where the remote HUB (RHUB) is installed; and (iii) the production area where the pRRUs are scattered in order to increase the covered area. The BBU, RHUB, and pRRUs are used to compose the RAN solution in the 5GAIner network.

The pRRUs distribution was decided with the objective of maximising coverage of the shop floor while ensuring that the use case areas have network coverage.

The network is owned by ITAV, and the spectrum used is a reserved band of a Portugal operator called Altice. The 5GAIner infrastructure is currently running a Release 15 (R15) 5G network solution, and Table 2 presents the radio configurations.

All the above information of the 5G network deployed allows for the understanding of the current performance metrics and network limitations. Additionally, an R15 5G network only supports eMBB slices. Therefore, in the results presented in this paper, eMBB is considered as a default configuration of the 5G network.

### 4.3. Intelligent Assistant

Data collected on the shop floor are expected to enable real-time and retrospective access to the working conditions and operation of industrial assets and their surroundings. The availability of this type of information makes it possible to create applications that can analyse and use it for intelligent monitoring, maintenance, and/or management of assets on the shop floor.

**Figure 6:**
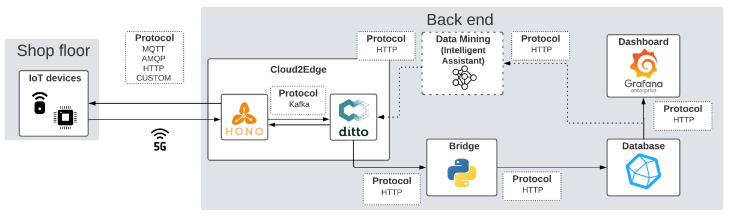
Bosch TT’s data management framework (back-end component deployed in a local server).

Within the scope of a data management framework, Bosch TT defined the data pipeline shown in Figure 6, where the goal is to communicate shop floor data to back-end operations for data storage, processing, and mining, thus enabling data-driven and intelligent decision making. The pipeline is an evolution of the smart cloud of things (SCoT) [34], resulting from different research works in the scope of the Augmanity project at Bosch TT [35,36,37].

Shop floor data are sent to the back-end via MQTT over the 5G network. The messaging platform with the back-end is the open-source Eclipse IoT Cloud2Edge package (https://www.eclipse.org/packages/packages/cloud2edge/), with Eclipse Hono handling data exchanges and device registries, while Eclipse Ditto manages virtual replicas (digital twins) of the devices and pre-processes their data. The Cloud2Edge package is deployed at the edge within the factory premises and is only accessible through the industrial 5G network, ensuring that data are confined to the industrial premises.

A bridge service taps into the incoming digital twin data updates from Eclipse Ditto and forwards the equipment status to InfluxDB (https://www.influxdata.com/), a time-series database, which is optimal for IoT scenarios. A Grafana (https://grafana.com/) instance is also deployed to display data stored in the time-series database in a user-friendly way.

This data pipeline enables facilitated monitoring, storage, and access to the assets and their data, which can be used for fault prediction and anomaly detection aiming at predictive maintenance and energy management use cases.

**Figure 7:**
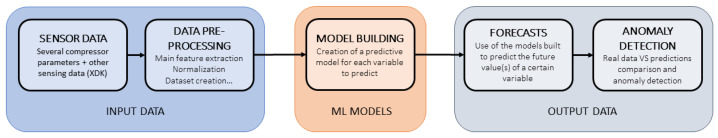
Proposed architecture for the intelligent assistant component.

In that regard, our application component intelligent assistant [38] (cf., Figure 7) is proposed for forecasting the asset’s time-series data evolution and for detecting possible anomalies.

As illustrated in Figure 7, sensor data reach the intelligent assistant through the data pipeline presented in Figure 6. It is then pre-processed with operations such as normalisation (to values between 0 and 1) or time-series aggregation (grouping datapoints minute by minute instead of second by second).

The resulting data are then used to build and train the ML models. The building process is run with the help of automated machine learning (AutoML), which allows for a more flexible building process: first, it selects an appropriate model that suits the data, and then, it can manipulate its hyperparameters, through a series of iterations, to reach an optimised model for the specific variable to predict.

With the models built and stored, the intelligent assistant can (i) make predictions for each variable being monitored regarding their future values, and (ii) detect anomalies by comparing them to the predictions and the defined thresholds.

**Figure 8:**
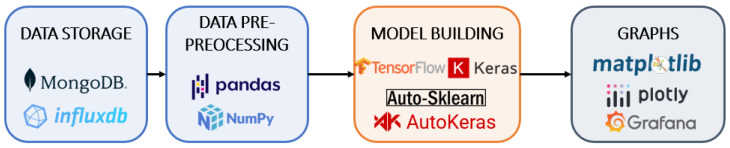
Implementation of the intelligent assistant component.

The intelligent assistant and respective algorithms were implemented based on Python, and other tools are shown in Figure 8.

The data gathering is performed via two time-series databases—MongoDB and InfluxDB—with PyMongo 4.0.1 and InfluxDBClient 1.29.0 being the two Python libraries used to transfer the data. The processing of the data is performed mainly with the aid of Pandas 1.4.1 and NumPy 1.22.2 frameworks, widely used for data management, processing and analysis. Then, to build, train and implement the models, libraries such as Tensorflow, Auto-Sklearn or AutoKeras were extensively used. Finally, the visualisation of data was first accomplished with Python libraries such as MatPlotLib 3.5.1 or Plotly 5.6.0, and then, with the intelligent assistant running, through Grafana dashboards.

## 5. Validation Tests

As mentioned in Section 4, despite our IIoT ecosystem over a 5G NPN being complete, we are yet to deploy and test its full-fledged version in production. Thus, this section presents a set of validation tests that helped assess the implementation and operation of our proposed infrastructure and application components on the shop floor but not yet in production.

### 5.1. Capability Assessment of the Industrial 5G Network

**Figure 9:**
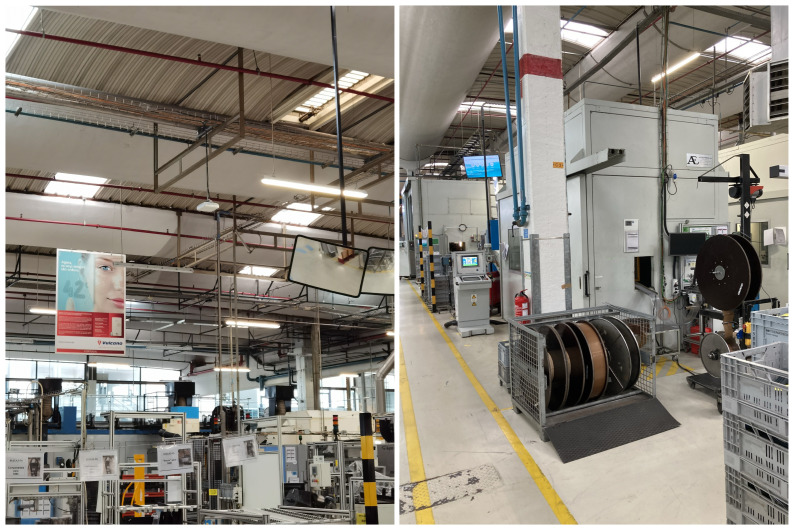
Environment of Bosch TT factory: heavy presence of metal (e.g., tools, framing, casings, roof) which may affect antenna’s coverage.

The deployed solution uses a licensed spectrum provided by the network operator; thus, interference is not considered to be an issue in the validation process, and no indication of interference effects was found during the evaluations. However, the factory environment contains a huge amount of metal distributed throughout the shop floor (as shown in Figure 9). Due to the uncertainty that the antenna’s coverage would be affected by the metal, we initially deployed the antennas in more adjacent locations than the ones presented in Section 4.2. Then, several coverage tests were made to examine the performance and coverage on the shop floor. The test results were used to decide the current deployment locations that significantly improved the network in terms of coverage.

A coverage test was conducted with the current deployment to check the coverage and to know the performance in each shop floor location. This coverage test contains a set of evaluations that analyse the latency, reference signal received power (RSRP), coverage, and throughput. The reasons for these tests are twofold,: on the one hand, it provides the coverage and expected performance at each location, and on the other hand, it allows for evaluation of the deployment topology of the network, which on the initial deployment was used to relocate the antennas to a better placement.

**Figure 10:**
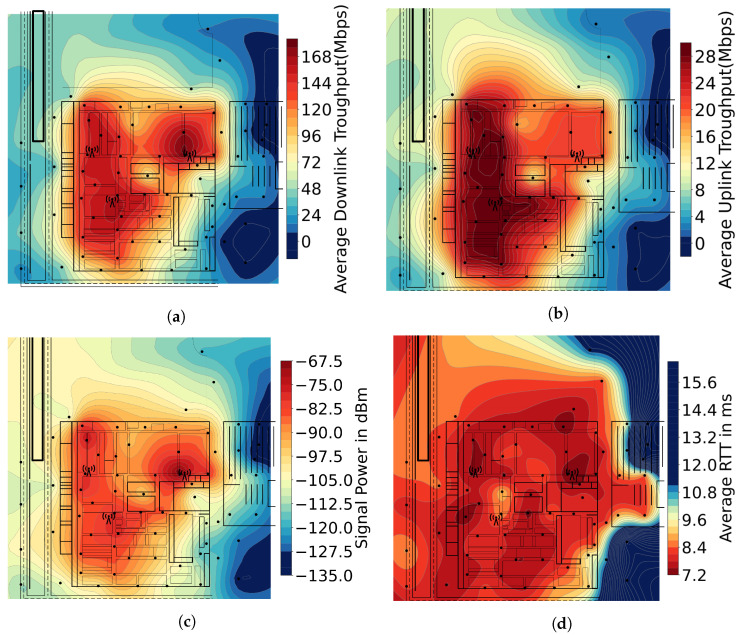
Performance of 5G network in the factory premises. (**a**) Mean downlink. (**b**) Mean uplink. (**c**) RSRP. (**d**) Average ping.

Figure 10 presents the results obtained in the tests. The black lines represent the shop floor and nearby routes, while the black dots are the locations where the measurements took place, and the antenna symbols are the actual antenna locations. For these tests, we used a Huawei P40 Pro with the termux (https://termux.dev/en/) app installed.

To obtain the values for Figure 10a,b, we used iperf3 (https://iperf.fr/) running for one minute with user datagram protocol (UDP) with no bandwidth limit at each measurement point for uplink and for downlink, and we used the measures obtained by the 5G network metrics to produce the graphs. For the values presented in Figure 10c, the RSRP values that the mobile phone registered in each location were used. To obtain the values of Figure 10d, we used ping, sending one ping every 200 ms with a total of 31 pings. Then, to produce the graphs shown in Figure 10, we used Universal Kriging interpolation with the spherical method to obtain an estimated network performance value in the entire shop floor.

The results show that most shop floor areas have a downlink throughput above 100 Mbps, an uplink throughput above 16 Mbps, a RSRP below −90 dBm, and an average round-trip time (RTT) below 9 ms. One can observe that the shop floor is mostly covered, which gives extreme flexibility and freedom for deploying, testing, and evaluating different use cases on the entire shop floor.

### 5.2. Performance Assessment of the 5G IoT End Device

For the performance assessment of the 5G IoT end device, we considered our proposed industrial 5G network and tested latency, packet loss, energy consumption, and throughput. To validate the latency and packet loss, we used ping, sending a packet every second for one hour from the end device to the MEC, where the communication RTT values were registered. To validate throughput and energy consumption, the end device was configured to communicate with an MQTT server, sending all the information collected from the sensors every second for one hour, thus simulating the data flow of the use cases presented in Section 3. The end device throughput was measured using the metrics collected by the 5G network, and the energy consumption was measured using a Keithley 2460 source meter unit (SMU) (https://www.tek.com/en/datasheet/2460-source-measure-unit).

During the development phase of the end device, we noticed that the communication between the SoC and the 5G modem could increase the latency times due to the reduced throughput that the UART communication provided. Since the modem communicates with the SoC via UART, the baud rate should limit the transmission speed. Following the RG502 datasheet [31], the default value for the UART baud rate was 115200 and the support for different baud rates was under development. Considering the start and stop bit, this means that the maximum theoretical throughput would be 92 kbps. These rates could result in a bottleneck not only for throughput but also for latency.

To measure the impact caused by the UART on the latency, the modem was connected with two protocol data unit (PDU) sessions, where (i) the PDU session is connected to the 5G modem and does not transmit packets to the SoC; and (ii) the PDU session is connected to the SoC through the 5G modem via UART. Then, ping sends a packet every second from the MEC to each PDU session for one hour. The RTT was measured for both the 5G modem and the SoC sessions at different moments.

**Figure 11:**
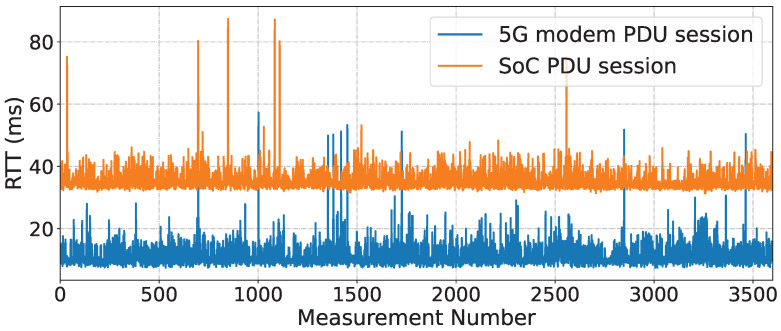
RTT values for the 5G modem and SoC sessions.

Figure 11 presents the obtained RTT values, with the mean RTT value of communication to the 5G modem at 11.32243 ± 3.81537 ms and to the SoC at 35.34099 ± 3.20677 ms. This 24 ms difference shows that UART communication between the SoC and the 5G modem causes a significant increase in the measured latency.

For the latency test, we considered three network configurations with the SoC PDU session: (1) eMBB—which uses the default configurations of the 5GAIner network as mentioned in Section 4.2; (2) latency—in which we optimised several functionalities to reduce latency by adapting the network configuration to the simulated use cases, increasing pre-allocation duration, reducing the time between scheduling slots, increasing the transmission priority and having reserved bandwidth for transmission; and (3) energy—aimed at reducing energy consumption, where we disabled pre-allocation, increased the time between scheduling slots, reduced the time that the end device was monitoring the downlink control channel, allowing the antennas to enter sleep mode, and disabled single-user MIMO (multiple-input/multiple-output).

**Figure 12:**
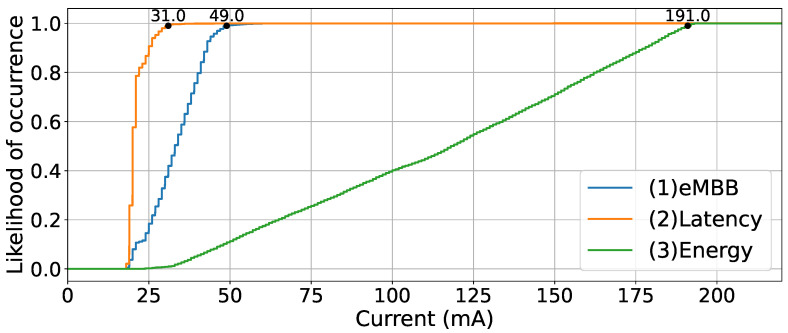
CDF of RTT values with the three different network configurations validated.

As for the packet loss, all packets were transmitted successfully in the tests for all network configurations from the 3600 pings sent. Figure 12 presents the cumulative distribution function (CDF) of the obtained RTT values. The 99th percentile value is 31 ms for (1) eMBB, 49 ms for (2) latency, and 119 ms for (3) energy, as shown in the graph. As expected, the best RTT values happen when the network is optimised for reducing latency. The (2) latency configuration reduces RTT due to having a pre-allocation adjusted to the transmission periodicity, which ensures an allocated slot at the moment of transmission. In addition, this configuration has higher transmission priority, lower time between slots used for requesting transmission resources, and reserved bandwidth for transmission. On the other hand, the (3) energy configuration imputes longer delay to the communication since there is no pre-allocation, the time between slots used for requesting transmission resources is higher, and the 5G modem can be in sleep mode at the exact moment the transmission starts, requiring the antennas to be activated before requesting the transmission.

**Figure 13:**
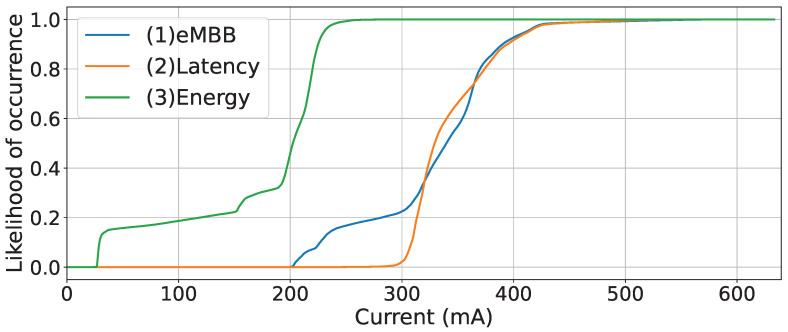
CDF of current consumption with the three different network configurations validated.

For the throughput and energy-consumption tests, the end device was powered via a USB-C with a voltage of 5.2 V and was tested for the three network configurations used in the latency test using the SoC PDU session. Regarding the throughput, the end device sent around 1510 bits per second (bps) in the uplink and received around 1030 bps in the downlink from the MQTT server regardless of the network configuration. The current consumption during the tests is presented in Figure 13 for each network configuration.

The mean current consumption values are 328.7 ± 60.6 mA for (1) eMBB, 342.8 ± 37.2 mA for (2) latency, and 172.1 ± 69.5 mA for (3) energy. The (2) latency configuration slightly increases energy consumption when compared to the (1) eMBB configuration. This is mainly due to the increase in pre-allocation duration, which makes the end device transmit more packets to the network. In addition, there is a significant difference in the current used by the end device in the (3) energy configuration in comparison to the (1) eMBB and (2) latency configurations. This was expected since the end device was not monitoring the downlink control channel all the time, being allowed to sleep between these events and to save energy while doing so. In addition, disabling pre-allocation reduced the number of packets sent by the end device, thus reducing energy consumption.

The initial tests proved the impact that the SoC modem communication can have on latency. Even with this latency increase, the performance improvement in terms of latency compared to previous generations of cellular networks was still notable. The assessment tests for latency in the (1) eMBB configuration corresponds to the default network configuration of 5GAIner. This configuration is used to understand the end-device performance in a 5G network and is used as a reference performance. The (2) latency configuration results were satisfactory if we take into account the UART limitation and the network release used, while the results for the (3) energy configuration had an acceptable performance in terms of latency, considering the overall use case. The end device’s energy consumption was high for an IoT device, but this was a known compromise of using a non-optimised 5G NR modem and a network release that does not support massive machine-type communications (mMTC) slices. That said, while the consumption for both (1) eMBB and (2) latency configurations was intensive, the reduction in energy consumption for the (3) energy configuration was significant, and this configuration would be the most adequate considering the particularities of the use cases presented.

### 5.3. Intelligent Assistant

The intelligent assistant was validated considering its ability to make correct predictions and to detect anomalies correctly. The asset was an industrial air compressor. Regarding the predictions, the root mean squared error (RMSE) was used to evaluate the models’ quality numerically.

**Figure 14:**
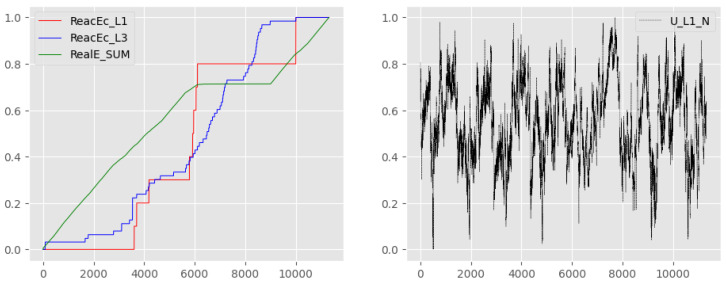
Linear behaviour of the variables ReacEc_L1, ReacEc_L3, and RealE_SUM when compared to the non-linear behaviour of U_L1_N.

Depending on the number of future timestamps to predict (T), the RMSE would calculate the error between the real values and the predictions. The smaller the RMSE, the better the prediction. Additionally, any AutoML model with higher RMSE values than a linear regression model would be discarded. That was the case for the ReacEc_L1, ReacEc_L3, and RealE_SUM variables, which had a much more linear behaviour when compared with the remaining variables. As it can be seen in Table 3 and Figure 14, the three variables mentioned above (ReacEc_L1, ReacEc_L3, and RealE_SUM), given their linear behaviour, are better suited by a linear regression model.

In relation to the two AutoML libraries, the models generated using AutoSKLearn outperform those constructed with AutoKeras. This indicates that the time-series data acquired from the compressor are better represented by an ensemble of models created by the AutoSKLearn library, which combines different algorithm characteristics to construct an optimised model. On the other hand, the deep learning approach employed by the AutoKeras library is not as effective in this case (more details available in [38]). Furthermore, in addition to the three aforementioned variables that exhibit more linear behaviour (ReacEc_L1, ReacEc_L3 and RealE_SUM), the AutoSKLearn models demonstrate enhancements in terms of the obtained RMSE.

To analyse the models’ performance regarding anomaly detection, two kinds of faults were generated in the datasets to simulate (1) a sharp and abrupt decline in a variable’s value, and (2) a prolonged power outage/energy spike. Then, some metrics were used to numerically showcase their performance: the calculation of the number of false and true negatives and positives (confusion matrix) as well as the precision, recall, and F1 score. The results of the analysis are gathered in Table 4 and Table 5.

Comparing the results between the two types of anomalies, the recall was much higher regarding the point anomaly detection. That means that the models could correctly identify the point anomalies, as there were only zero or one false negatives (FN), i.e., only one falsely identified anomaly. On the other hand, for the “prolonged” anomalies, some models showcased very low recalls, revealing that the models tended to learn that the anomalies come majorly in the form of spikes and that they predicted that the normal behaviour would come shortly after the anomaly occurred.

Looking at the precision, the behaviour was completely the opposite, as the models showcased larger precision in the datasets with longer anomalous periods. When there were point anomalies in the datasets, the models tended to predict way more anomalies than the ones that actually existed (for instance, for the ReacEc_L1 variable, in Table 4, there were 50 false positives, or 50 points falsely considered as anomalies, contrasting to the 9 false positives shown in Table 5, relative to longer anomalous periods).

Finally, regarding the F1 score, which represents the harmonic mean between recall and precision, models such as the H_TDH_U_L2_N or the P_SUM revealed a really good performance, meaning that they were very well suited for the anomaly detection task, independent of the type of anomalies. On the other hand, for instance for the ReacEc_L1 variable, the low F1 score indicates that the model needed to be further trained in order to be able to correctly predict the anomalies, or even that the threshold from which a point was considered an anomaly needed to be changed.

## 6. Conclusions

While 5G NR technology holds great potential for supporting the connectivity needs of embedded devices in industrial applications, there are currently some limitations to its adoption. The current 5G modems are not optimised for low-power-constrained devices, which can significantly hinder their implementation in certain use cases. In addition, the lack of built-in stack support for the transport and above layers of the considered RG502 forced us to make the SoC-modem communication over UART to support AT commands. This meant a performance reduction in both throughput and latency, but it was a necessary compromise if we wanted to have 5G NR in an embedded solution. Even though the results are not representative of what 5G NR can bring to the industry, they were sufficient for the use case requirements in this work, accomplishing the goal of practically implementing the proposed 5G IoT end device using 5G NR.

The market is expected to evolve with the development of more energy-efficient 5G modems better suited for low-power-constrained devices. While the development of the 5G IoT end device was performed with 5G NR in mind, both the hardware and firmware were implemented with abstraction from the 5G NR modem itself, and it is fairly easy to develop future solutions not only using 5G NR but narrow band IoT (NB-IoT), long-term evolution machine-type communication (LTE-M), or even recent technologies such as RedCap [39] if the use case requires. New devices have been released during the course of this project such as the RG225C from Quectel [40], highlighting the continued innovation in the market for 5G modems. If the IoT market evolves towards 5G, we can expect modems with SPI peripheral support so that a connection to another device can be established with higher data rates.

A 5G network released in R15 does not provide everything expected to be available in 5G, but as shown in this document, it is already able to support industry 4.0 use cases. Additionally, a 5G network is said to have great performance in many communication metrics, such as latency, connected users, energy consumption, and others. However, as demonstrated in the validation, it is not possible to maximize the performance of all communication metrics at the same time. Where it was shown that when the network is optimised for latency the energy consumption increases, and when optimised for energy consumption, there is an increase in the latency. Therefore, when deciding the communication requirements for an industrial use case, it is necessary to define the most critical requirements to make the trade-offs necessary to achieve the desired communication performance.

The performance results obtained with the different network configurations in the previous section demonstrate that since the R15 of the 5G network, it is possible to adjust the performance of the communications to improve specific performance parameters, showing that it is possible to achieve a performance closer to the one expected to be available in an mMTC and a ultra-reliable low-latency communications (URLLC) slice, in comparison to the performance available in an eMBB slice. With the rolling out of higher releases, some current functionalities will have higher control granularity, and new network functionalities and parameters will become available, making it possible for better and more granular adjustments to the communication performance.

Finally, an IIoT ecosystem over 5G, presented in this work, serves as the basis for the implementation of an assistant capable of processing the data. The proposed 5G infrastructure components coupled with Bosch TT’s data management framework allow for the collection and storage of large amounts of factory data (e.g., air quality, vehicle travel routes, noise, movements, energy consumption, location, among others), which the intelligent assistant can then process.

The implemented assistant revealed good results, especially regarding making precise predictions and detecting anomalies. However, this proposed assistant still has much potential to be uncovered, as it can have a much larger impact on improving process efficiency on the shop floor. For instance, the results of the anomaly detection can be further processed to generate automated, real-time warnings transmitted to production workers, maintenance teams, etc. The assistant can also use this information to automatically generate daily, weekly, or shift reports to teach itself and to further enhance its capabilities. Thinking even further, the assistant could automate some processes by sending back commands, through the 5G infrastructure components, to actuate (i.e., change) the operational status of the physical industrial assets when an anomaly or an event is detected.

This practical implementation of the proposed infrastructure and application components is still evolving within the context of the Augmanity project. More industrial assets are expected to be monitored, and different applications shall emerge to improve processes on the shop floor further while targeting a smarter, more sustainable, green, and environmentally friendly industry.

## Figures and Tables

**Table 1:** Main requirements targeted by the proposed infrastructure and application components.

Component	Retrofit and Predictive Maintenance	Energy Management
5G IoT end device	5G NR support	
	Multiple sensing capabilities	
	Low energy consumption	
	Modular and retrofittable	
	MQTT support	
	Actuate on industrial assets	
	Protective and easily accessible enclosure	
Industrial 5G Network	Guaranteed Throughput	High density of devices
	Low energy consumption	
	Reliable communication	
	Industrial data needs to remain in factory premises	
	Industrial MEC needs to be logically isolated from the public network	
	All industrial communications need to be secure	
Intelligent Assistant	Fault prediction	
	Anomaly detection	
	Alarm/report creation	

**Table 2:** gNodeB technical specifications.

Specification	Values
Maximum bandwidth	20 MHz with 30 kHz subcarrier spacing
Frequency band	Centre frequency of 3790.02 MHz (n78 band)
Output power per port	24 dBm
Demultiplexing	TDD
DL Modulation	BPSK; QPSK; 16/64/256QAM
UL Modulation	BPSK; QPSK; 16/64QAM
MIMO	4T4R
Network Slicing	eMBB Slices
Slot assignment	4 Downlink: 1 Uplink (Slot structure 2)

**Table 3:** RMSE values from three different models for the variables to predict.

Model	Linear Regression	AutoSKLearn	AutoKeras
C_phi_L3	0.055232	0.044430	0.052167
F	0.040205	0.039560	0.041842
H_TDH_I_L3_N	0.046060	0.034610	0.050411
H_TDH_U_L2_N	0.030794	0.030430	0.032300
I_SUM	0.065990	0.057850	0.069731
P_SUM	0.079265	0.063210	0.144370
ReacEc_L1	0.000090	0.014230	0.098168
ReacEc_L3	0.000133	0.004760	0.188699
RealE_SUM	0.000004	0.044310	0.340505
U_L1_N	0.031372	0.030960	0.031636

**Table 4:** Metrics results for the anomaly detection with random point spikes applied to the datasets.

Model	TP	FP	TN	FN	Recall	Precision	F1 Score
C_phi_L3	26	26	3888	1	0.962963	0.500000	0.658228
F	26	26	3888	1	0.962963	0.500000	0.658228
H_TDH_I_L3_N	26	10	3904	1	0.962963	0.722222	0.825397
H_TDH_U_L2_N	27	1	3913	0	1.000000	0.964286	0.981818
I_SUM	26	26	3888	1	0.962963	0.500000	0.658228
P_SUM	26	6	3908	1	0.962963	0.812500	0.881356
ReacEc_L1	27	50	3860	0	1.000000	0.350649	0.519231
ReacEc_L3	26	26	3888	1	0.962963	0.500000	0.658228
RealE_SUM	27	326	3588	0	1.000000	0.076487	0.142105
U_L1_N	27	322	3592	0	1.000000	0.077364	0.143617

**Table 5:** Metrics results for the anomaly detection with simulated long power outages in the datasets.

Model	TP	FP	TN	FN	Recall	Precision	F1 Score
C_phi_L3	37	6	3737	161	0.186869	0.860465	0.307054
F	173	8	3735	25	0.873737	0.955801	0.912929
H_TDH_I_L3_N	157	0	3743	41	0.792929	1.000000	0.884507
H_TDH_U_L2_N	180	1	3742	18	0.909091	0.994475	0.949868
I_SUM	179	3	3740	19	0.904040	0.983516	0.942105
P_SUM	182	6	3737	6	0.919192	0.968085	0.943005
ReacEc_L1	27	9	3734	171	0.136364	0.750000	0.230769
ReacEc_L3	18	0	3743	180	0.090909	1.000000	0.166667
RealE_SUM	193	123	3620	5	0.974747	0.610759	0.750973
U_L1_N	191	164	3579	7	0.964646	0.538028	0.690778

## Data Availability

Not applicable.

## Abbreviations

The following abbreviations are used in this manuscript:

3GPP	Third Generation Partnership Project
5G-ACIA	5G Alliance for Connected Industries and Automation
5G SA	5G Standalone
5G NSA	5G Non Standalone
5G NR	5G New Radio
AUSF	Authentication Server Function
AMF	Access and Mobility Management Function
BBU	Baseband Unit
BLE	Bluetooth Low Energy
Bosch TT	Bosch Termotecnologia
CDF	Cumulative Distribution Function
CPE	Customer-Premises Equipment
DNN	Data Network Name
eMBB	Enhanced Mobile Broadband
GPIO	General Purpose Input/Output
GSM	Global System for Mobile Communications
I^2^C	Inter-Integrated Circuit
IIoT	Industrial Internet of Things
IoT	Internet of Things
IP	Internet Protocol
ITAV	Instituto de Telecomunicações in Aveiro
KPI	Key Performance Indicator
LADN	Local Area Data Network
LBO	Local BreakOut
LTE	Long-Term Evolution
LTE-M	Long-Term Evolution Machine Type Communication
MEC	Multi-access Edge Computing
ML	Machine Learning
mMTC	Massive Machine-Type Communications
MQTT	Message Queuing Telemetry Transport
NB-IoT	Narrow Band IoT
NFs	Network Functions
NFVI	NFV Infrastructure
NPN	Non-Public Network
NRF	Network Repository Function
NSSF	Network Slice Selection Function
PCB	Printed Circuit Board
PDU	Protocol Data Unit
PNI-NPN	Public Network Integrated Non-Public Network
PPP	Point-to-Point Protocol
pRRU	Pico Remote Radio Unit
R15	Release 15
RAN	Radio Access Network
RGB	RGB
RHUB	Remote HUB
RMSE	Root Mean Squared Error
RSRP	Reference Signal Received Power
RTT	Round-Trip Time
SCoT	Smart Cloud of Things
SIM	Subscriber Identity Module
SMF	Session Management Function
SMU	Source Meter Unit
SNPN	Stand-Alone Non-Public Network
SoC	System-on-Chip
SPI	Serial Peripheral Interface
TA	Tracking Area
TAI	Tracking Area Identifier
TCP	Transmission Control Protocol
UART	Universal Asynchronous Receiver–Transmitter
UDM	Unified Data Management
UDP	User Datagram Protocol
UE	User Equipment
ULCL	Uplink Classifier
UPF	User Plane Function
URLLC	Ultra-Reliable Low Latency Communications
USB	Universal Serial Bus
USB-C	Universal Serial Bus Type-C
